# Proximal Tubule‐Specific Genetic Deficiency of PPARα Worsens Systemic Lipid and Glucose Metabolism During Fasting

**DOI:** 10.1096/fj.202502975R

**Published:** 2025-12-29

**Authors:** Daiki Aomura, Takayuki Nimura, Kosuke Yamaka, Yosuke Yamada, Makoto Harada, Takero Nakajima, Takefumi Kimura, Koji Hashimoto, Hiroyuki Kanno, Motoko Yanagita, Frank J. Gonzalez, Naoki Tanaka, Yuji Kamijo

**Affiliations:** ^1^ Department of Nephrology Shinshu University School of Medicine Matsumoto Japan; ^2^ Department of Metabolic Regulation Shinshu University School of Medicine Matsumoto Japan; ^3^ Center for Medical Education and Clinical Training, Shinshu University School of Medicine Matsumoto Japan; ^4^ Department of Gastroenterology Shinshu University School of Medicine Matsumoto Japan; ^5^ Department of Pathology Shinshu University School of Medicine Matsumoto Japan; ^6^ Department of Nephrology Kyoto University School of Medicine Kyoto Japan; ^7^ Institute for the Advanced Study of Human Biology (ASHBi), Kyoto University Kyoto Japan; ^8^ Laboratory of Metabolism, Center for Cancer Research, National Cancer Institute, National Institutes of Health Bethesda USA; ^9^ Department of Global Medical Research Promotion Shinshu University Graduate School of Medicine Matsumoto Japan; ^10^ International Relations Office, Shinshu University School of Medicine Matsumoto Japan; ^11^ Research Center for Social Systems, Shinshu University Matsumoto Japan

**Keywords:** fasting, fatty acids, gluconeogenesis, ketone bodies, kidney tubules, lipolysis, PPAR alpha, proximal

## Abstract

The proximal tubule (PT) of the kidney is a highly metabolic organ that regulates systemic homeostasis through ketogenesis and gluconeogenesis. Peroxisome proliferator‐activated receptor alpha (PPARα), a nuclear receptor controlling fatty acid oxidation (FAO) and homeostasis, is expressed in the PT and is activated by fasting. Although the activation of systemic PPARα is essential for renal and systemic energy metabolism, the role of PPARα in PT has not been established. In this study, kidney PT‐specific PPARα knockout mice (*Ppara* ^∆KPT^) were generated, and the metabolic changes caused by 48 h of fasting were compared between *Ppara* ^∆KPT^ mice and controls. In *Ppara* ^∆KPT^ mice, renal FAO and ketogenesis were severely impaired, leading to lipid accumulation in the kidney after 48 h of fasting. The increase in the renal expression of gluconeogenesis‐associated genes due to fasting was insufficient in *Ppara* ^∆KPT^ mice, causing a decrease in serum glucose levels and liver glycogen content. Fasting caused hepatic micro‐steatosis and significantly increased the expression of genes linked to FAO and ketogenesis in the liver of *Ppara* ^∆KPT^ mice, as well as those associated with lipolysis in the white adipose tissue. These activities likely compensate for the impaired kidney FAO and ketogenesis in *Ppara* ^∆KPT^ mice and show that PPARα in PT regulates renal and systemic lipid and glucose metabolism during fasting. PPARα in PT may be a critical component in systemic lipid and glucose homeostasis during fasting.

## Graphical abstract

Proximal tubule‐specific PPARα knockout mouse (PPARα^ΔKPT^) showed decreased lipid and glucose metabolism in the kidney during fasting. Metabolism in the other organs was increased, presumably as a means of compensating for the impaired kidney metabolism. PPARα in the proximal tubule plays a paramount role in renal and systemic lipid and glucose metabolism during fasting.

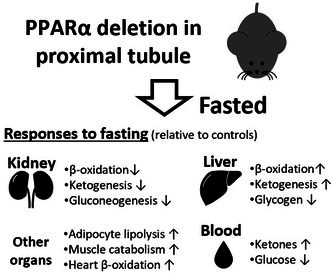

**Abbreviations**
ATP: adenosine triphosphate
BW: body weight
CKD: chronic kidney disease
CPT2: carnitine palmitoyltransferase 2
CT: computed tomography
ESRRα: estrogen‐related receptor alpha
eWAT: epididymal white adipose tissue
FAO: fatty acid oxidation
FGF21: fibroblast growth factor 21
G6PC: glucose 6‐phosphatase
GAPDH: glyceraldehyde 3‐phosphate dehydrogenase
Global Ppara−/−: global PPARα knockout mice
GLUTs: glucose transporters
GM: gastrocnemius muscle
GNG: gluconeogenesis
GTT: glucose tolerance test
HE: hematoxylin and eosin
HMGCS2: 3‐hydroxy‐3‐methylglutaryl‐coenzyme A synthase 2
HNF4α: hepatocyte nuclear factor 4‐alpha
ITT: insulin tolerance test
LDHA: lactate dehydrogenase A
mTOR: mammalian target of rapamycin
NEFA: nonesterified fatty acids
NIH: National Institutes of Health
PAS: periodic acid‐Schiff
PCR: polymerase chain reaction
PEPCK: phosphoenolpyruvate carboxykinase
PGC1α: peroxisome proliferator‐activated receptor gamma coactivator 1‐alpha
Ppara∆HEP: liver‐specific Ppara‐null mice
Ppara∆KPT: proximal tubule‐specific PPARα‐knockout mice (Ndrg1CreERT2/+‐Pparafl/fl)
Pparafl/fl controls: Pparafl/fl mice
PPARα: peroxisome proliferator‐activated receptor α
PT: acetyl‐CoA acyltransferase
PT: proximal tubule
RXRα: retinoid X receptor alpha
TBP: TATA box‐binding proteins
TG: triglyceride
TM: tamoxifen
VLCAD: very long‐chain acyl‐CoA dehydrogenase
β‐OHB: β‐hydroxybutyrate

## 1 Introduction

The proximal tubule (PT) in the kidney regulates fluid, electrolyte, and nutrient homeostasis by reabsorption of filtered water and solutes from the glomeruli [1]. Additionally, PT contributes to systemic energy metabolism via ketogenesis and gluconeogenesis (GNG) [2, 3, 4]. PT consumes a massive amount of adenosine triphosphate (ATP) to maintain these functions, and its energy supply is mostly due to fatty acid oxidation (FAO) [5, 6, 7]. FAO in PT plays a central role in the homeostasis of renal energy metabolism, and its activation is expected to be renoprotective against various kidney diseases [8].

Peroxisome proliferator‐activated receptor α (PPARα), a member of the nuclear receptor superfamily, governs the expression of genes involved in FAO [9]. PPARα is expressed in highly metabolic rate organs such as the liver, heart, and PT in the kidney and are highly activated by fasting and other conditions that demand energy production from FAO [10, 11, 12]. Previous studies using global PPARα knockout mice (*Ppara* ^−/−^) or systemic PPARα agonists revealed that PPARα regulates the energy metabolism and is essential for maintaining systemic lipid and glucose metabolism [9, 13, 14, 15, 16]. Fasted global *Ppara* ^−/−^ mice were reported to exhibit systemic decreased fatty acid cellular uptake and FAO resulting in severe hypoglycemia, hypoketonemia, hypothermia, and elevated plasma free fatty acid levels [9]. These metabolic changes were thought to be mainly due to insufficient gene upregulation associated with hepatic FAO and subsequent insufficient supply of substrates that can be metabolized by other tissues. Fasted global *Ppara* ^−/−^ mice also exhibited renal tubular protein reabsorption abnormality due to energy depletion [13]; however, the influence of PPARα depletion on systemic organs, including the liver, could not be excluded.

Given this background, we hypothesized that PPARα in PT plays a pivotal role in the homeostasis of renal and systemic lipid and glucose metabolism. To clarify this issue, we generated kidney PT‐specific PPARα‐knockout mice (*Ppara* ^∆KPT^) and evaluated the metabolic changes induced by fasting, a physiological PPARα‐activating condition.

## 2 Materials and Methods

### 2.1 Animals

**FIGURE 1:**
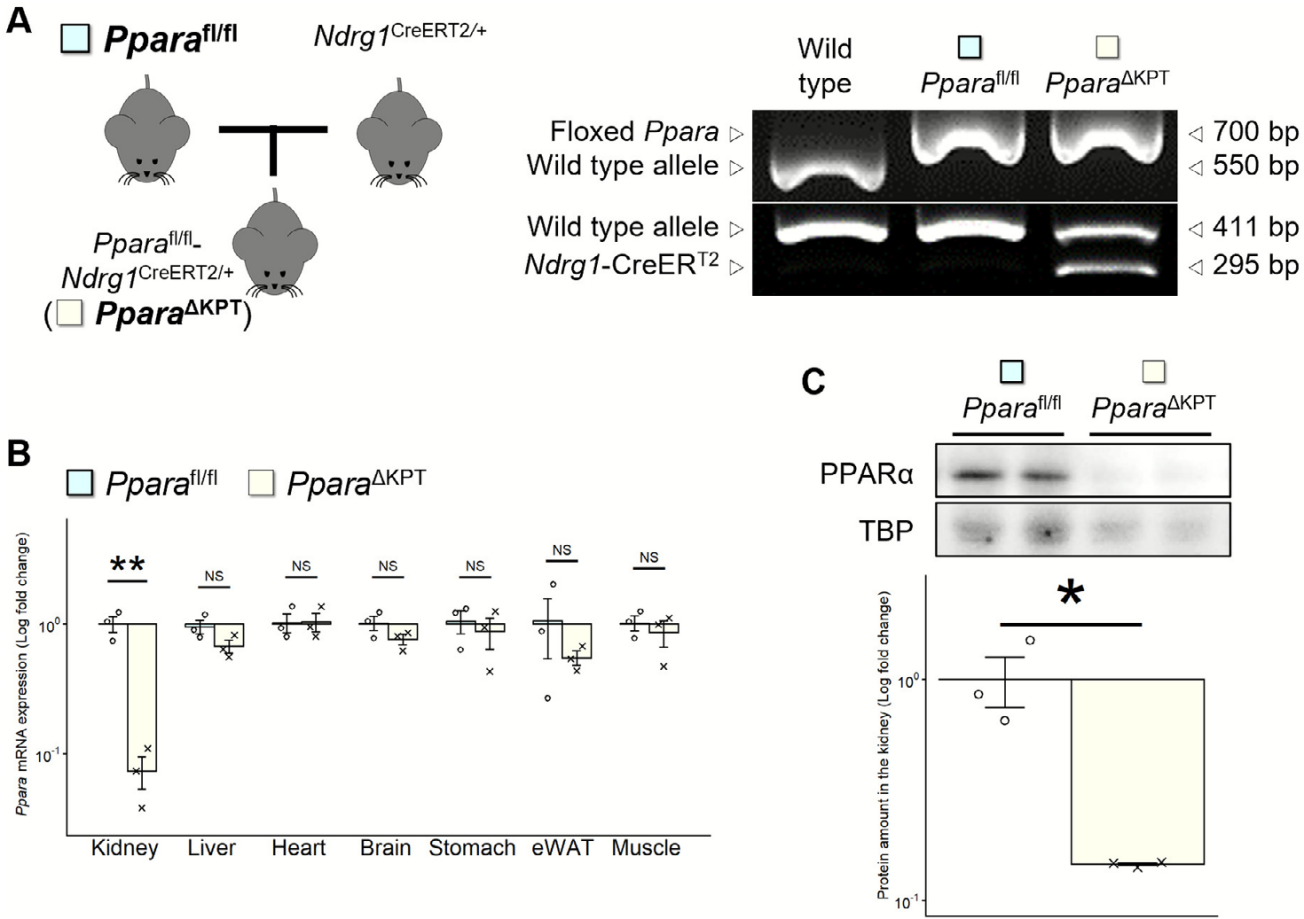
Confirmation of proximal tubule‐specific PPARα knockout in *Ppara* ^∆KPT^ mice. (A) Tamoxifen‐inducible and kidney proximal tubular specific‐PPARα knockout mice (*Ppara* ^∆KPT^) were generated by mating *Ppara* ^fl/fl^ with *Ndrg1* ^CreERT2/+^ mice. (B) mRNA expression of *Ppara* in the kidney and other organs. (C) Protein expression of PPARα in the kidney. Significant differences between the groups are indicated with asterisks. *: *p* < 0.05. **: *p* < 0.01. No significant differences are indicated with NS.

All animal procedures were approved by the Institutional Animal Care and Use Committee (IACUC) of Shinshu University School of Medicine (protocol codes 020111 and No. 17–106‐1), and the study was performed in accordance with the National Institutes of Health (NIH) Guide for the Care and Use of Laboratory Animals. All mice were maintained in a specific pathogen‐free facility, housed in a light‐ and temperature‐controlled environment (12‐h light/dark cycle at 25°C), and provided standard chow (except during fasting) and tap water ad libitum. To obtain PT‐specific PPARα knockout mice, we mated *Ndrg1* ^CreERT2*/+*^ with *Ppara* ^fl/fl^ on a C57BL/6J background [17, 18]. The *Ndrg1* gene is expressed in PT, and the efficacy of Cre recombinase in PT of *Ndrg1* ^CreERT2*/+*^ mice was reported to be 90% [17]. Male mice were genotyped as *Ndrg1* ^CreERT2*/+*^‐*Ppara* ^fl/fl^ (*Ppara* ^∆KPT^) or *Ppara* ^fl/fl^ by tail‐cut procedure and polymerase chain reaction (PCR) analysis (Figure 1A). Primer sequences used for genotyping are listed in Table S1. *Ppara* ^fl/fl^ was used as controls. *Ppara* ^∆KPT^ mice and controls at 8 weeks of age were intraperitoneally injected with 3 mg of tamoxifen (TM) (Cayman Chemical Company, MI, USA) for three consecutive days. TM was dissolved in a mixture of sunflower oil and ethanol in a ratio of 9:1. The mice were sacrificed under deep anesthesia with isoflurane 35 days after TM treatment (*n* = 3 vs. *n* = 3), and serum and organ samples were harvested from each animal. To confirm the validity of the TM treatment protocol, we also assessed *Ppara* ^∆KPT^ mice and *Ppara* ^fl/fl^ controls on day 35 after TM treatment for three or 5 days (*n* = 3 vs. 3, respectively), on day 21 or 49 after TM treatment for 3 days (*n* = 3 vs. 3, respectively), and without TM injection (*n* = 3 vs. 3). Next, *Ppara* ^∆KPT^ mice and *Ppara* ^fl/fl^ controls on day 35 after TM treatment for 3 days were fasted by depriving food in their cages for 0, 24, or 48 h (*n* = 7 vs. 5, 7 vs. 6, and 8 vs. 7, respectively). The mice not deprived of food were recognized as being under fed conditions. One *Ppara* ^∆KPT^ mouse and one *Ppara* ^fl/fl^ control were injured in fighting and excluded from the analyses. The mice were euthanized after each fasting period, and serum and organ samples were harvested. The gastrocnemius muscle (GM) and heart were harvested only from mice under fed conditions or fasted for 48 h. To collect urine from the mice, other *Ppara* ^∆KPT^ mice and *Ppara* ^fl/fl^ controls were fasted for 48 h in a metabolic cage (Tecniplast, Tokyo, Japan) (*n* = 4 vs. 4). Abdominal computed tomography (CT) images were obtained from other *Ppara* ^∆KPT^ mice and *Ppara* ^fl/fl^ controls (*n* = 3 vs. 3) before and after 48 h of fasting using LaTheta LCT‐200 (Hitachi Aloka Medical, Tokyo, Japan) to assess the body composition ratio. Mice were anesthetized with an intraperitoneal injection of medetomidine, midazolam, and butorphanol in each CT test. Fat mass and lean mass were estimated using the body fat measurement application in LaTheta LCT‐200. Insulin tolerance test (ITT) and glucose tolerance test (GTT) were performed using other *Ppara* ^∆KPT^ mice and *Ppara* ^fl/fl^ controls (*n* = 6 vs. 6, respectively). After a 16 h fast, the mice were intraperitoneally injected with insulin (0.5 units/kg BW, #101976, Eli Lilly Japan, Tokyo, Japan) for ITT or glucose (10 mg/kg BW) for GTT. The blood glucose levels were measured using LAB Gluco (#4239R1006, RIJ, Tokyo, Japan) at 0, 15, 30, 60, and 120 min postinjection.

### 2.2 Experimental Analyses

Blood tests were performed using a JCA‐BM6070 clinical analyzer (JEOL, Tokyo, Japan). Serum levels of insulin, glucagon, nonesterified fatty acid (NEFA), triglyceride (TG), and β‐hydroxybutyrate (β‐OHB) were measured using U‐type mouse insulin ELISA kits (Wako, Takasaki, Japan), glucagon ELISA kits (Wako, Takasaki, Japan), NEFA C‐test kits (Wako Pure Chemical, Osaka, Japan), triglyceride kits (Wako Pure Chemical, Osaka, Japan), and β‐HB Colorimetric Assay Kits (BioVision, Milpitas, CA, USA), respectively, according to the manufacturer's instructions. Urine tests, except for albuminuria tests, were performed using a JCA‐BM6050 clinical analyzer (JEOL, Tokyo, Japan). The urine albumin concentration was measured by immunoblotting as described below. Blood and urine tests were performed in a blind fashion with regard to sample information to minimize potential confounders. Total RNA was extracted from each organ sample using the RNeasy Mini Kit (QIAGEN, Hilden, Germany) according to the manufacturer's instructions. For total RNA extraction, the kidney was decapsulated, and the cortex was separated for analysis. During extraction from the gastrocnemius muscle and myocardium, 0.5 μg of Proteinase K (QIAGEN, Hilden, Germany) was added to the tissue homogenate, incubated at room temperature for 10 min, followed by heating at 50°C for 20 min. For the homogenates of epididymal white adipose tissue (eWAT) or cerebellum samples, 100 μL of chloroform was added, and RNA was extracted from the supernatant. RNA was reverse‐transcribed using oligo (dT) primers and SuperScript III reverse transcriptase (Invitrogen, Carlsbad, CA, USA). Subsequently, relative mRNA amounts were quantified by real‐time PCR using a THUNDERBIRD Probe qPCR Mix (TOYOBO, Osaka, Japan) on a QuantStudio 3 real‐time PCR system (Thermo Fisher Scientific, Waltham, MA, USA). *Actb* and *Tbp* genes were used as internal controls for cytoplasmic and nuclear genes, respectively. The details of the specific primers are listed in Table S2. The ΔΔCt method for PCR amplification was employed to calculate the relative expression of mRNA. Nuclear and cytoplasmic fractions of the tissue extracts were prepared using Nuclear and Cytosolic Extraction Reagents (Thermo Scientific, Rockford, IL, USA) with proteinase inhibitor cocktail (Thermo Scientific, Rockford, IL, USA) and PhosSTOP tablets (Roche, Indianapolis, USA). Equal amounts of protein were separated by sodium dodecyl sulfate‐polyacrylamide gel electrophoresis and incubated with primary antibodies, followed by incubation with horseradish peroxidase‐AffiniPure IgG (AB_10015289, Jackson ImmunoResearch Laboratories, Cambridge, UK). The detection of protein signals was carried out with a ChemiDoc Touch Imaging System (SCR_008426, Bio‐Rad Laboratories, Hercules, CA, USA) with EZ west Lumi plus (ATTO, Tokyo, Japan). Band intensities were measured densitometrically using ImageJ software version 1.54 (SCR_003070, NIH, Bethesda, MD, USA). β‐actin and TATA box‐binding proteins (TBP) served as internal controls for cytoplasmic and nuclear proteins, respectively. The expression of retinoid X receptor alpha (RXRα) and phospho‐mammalian target of rapamycin (p‐mTOR) was standardized based on the total protein amount assessed through Coomassie staining of the membrane. Urine albumin concentration was measured by immunoblotting. Urine samples were mixed with four times the volume of acetone and incubated at −20°C for 4 h. Extracted proteins were resolved in 2% sodium dodecyl sulfate‐sodium phosphate buffer. Immunoblotting was performed to estimate urine albumin concentration. Proteins extracted from murine blood, with albumin concentration determined using a JCA‐BM6070 clinical analyzer, were immunoblotted as standards for comparison with urine samples. The details of the primary antibodies are listed in Table S3. Total lipids were extracted from the kidney and liver using the hexane/isopropanol method, as previously described [19]. Briefly, 100 mg of kidney cortex or 30 mg of liver tissue was homogenized in 800 μL of ice‐cold distilled water. Lipids were extracted from 50 μL of homogenate using 900 μL of n‐hexane (3:2, v/v), dried in an evaporator, and weighed to determine the total amount of whole lipids extracted from the tissue. The dried lipids were dissolved in 100 μL of a 0.1% Triton X‐100 solution in ultrapure water, and NEFA and TG content were measured using NEFA C‐test kits and triglyceride kits, respectively. Murine tissues of the kidneys, liver, and eWAT were fixed in 10% formaldehyde for histological analyses. Deparaffinized sections were stained with periodic acid‐Schiff (PAS) or hematoxylin and eosin (HE). At the size assessment of adipocytes in eWAT, five HE stained images of nonoverlapping fields were randomly chosen, and the area of each adipose cell was automatically calculated using ImageJ software with the Adiposoft version 1.16 plug‐in [20]. Partial areas of harvested kidney and liver were acutely frozen, and lipids in the tissue were stained with Oil red O staining (Abcam, Cambridge, MA, USA) according to the manufacturer's instructions. Each stained section was observed under a CX41N‐31 microscope (Olympus, Tokyo, Japan).

### 2.3 Graph Creation and Statistical Analyses

In the figures, the value of each sample is described with bars representing the mean value (±standard error) of the group. The values of mRNA and protein expression are described on a log scale. Differences between the control and *Ppara* ^∆KPT^ groups at each assessment time point were statistically assessed using a two‐sided and unpaired Student's *t*‐test, and the values of *p* < 0.05 were considered statistically significant. The groups at different time points were not statistically compared. The area under the curve of serum glucose levels at ITT and GTT was calculated, and the difference between *Ppara* ^∆KPT^ mice and *Ppara* ^fl/fl^ controls was assessed using Student's *t*‐test. Graph creation and statistical analyses were performed using the R software (version 4.5.1, R Foundation for Statistical Computing, Vienna, Austria).

## 3 Results

### 3.1 *Ppara* ^∆KPT^ Mice Showed PT‐Specific PPARα Deletion

Real‐time PCR analysis revealed that TM‐treated *Ppara* ^∆KPT^ mice had 10% or less renal *Ppara* mRNA expression relative to *Ppara* ^fl/fl^ controls, regardless of the number of days of TM injection or time after TM treatment (Figure S1A,B). Both types of mice had residual oil in their abdominal cavity on day 35 after 5 days of TM treatment; however, all mice on day 35 after 3 days of TM treatment did not have residual oil. Therefore, additional assessments and experiments were performed using mice at day 35 after 3 days of TM treatment. *Ppara* mRNA expression in organs other than the kidney was similar between the genotypes (Figure 1B). Immunoblot analyses indicated that PPARα protein expression was completely depleted in the kidney of *Ppara* ^∆KPT^ mice (Figure 1C). Body weight (BW) was similar between the *Ppara* ^∆KPT^ and *Ppara* ^fl/fl^ control group (Figure S2).

### 3.2 Fasted *Ppara* ^∆KPT^ Mice Exhibited Higher Serum Ketone Levels and Lower Serum Glucose Levels

**FIGURE 2:**
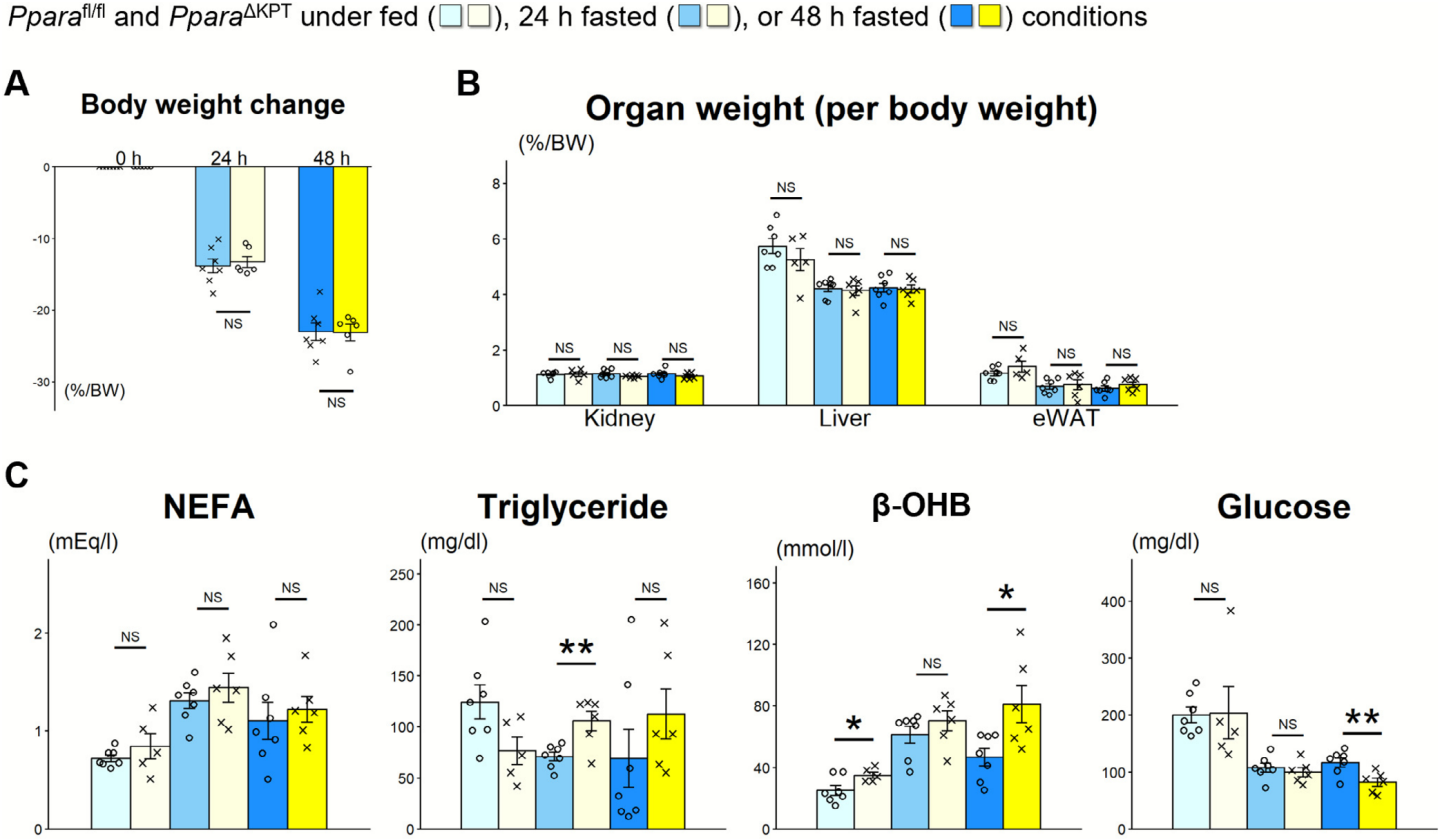
Body weight change and results of the blood test at the fasting experiment. *Ppara* ^∆KPT^ mice and *Ppara* ^fl/fl^ controls were sacrificed underfed conditions or after 24 or 48 h of fasting. (A) Decreased body weight during fasting. (B) Percentage of organ weight per body weight at each assessment point. (C) Results of the blood test. Significant differences between the *Ppara* ^∆KPT^ mice and *Ppara* ^fl/fl^ controls with the same fasting time are indicated with asterisks. *: *p* < 0.05. **: *p* < 0.01. No significant differences are indicated with NS.

During 48 h of fasting, BW, organ weight of the kidney, liver, eWAT, GM, and heart, and fat and lean mass weight were similarly decreased between *Ppara* ^∆KPT^ mice and *Ppara* ^fl/fl^ controls (Figures 2A,B, S3 and S4). Urine glucose levels after 48 h of fasting were significantly lower in *Ppara* ^∆KPT^ mice, and urine volume and urine albumin, N‐acetyl‐β‐D‐glucosaminidase, and electrolyte levels were not different between the mouse genotypes (Figure S5). Serum levels of TG after 24 h of fasting and serum levels of β‐OHB, a ketone body, under fed conditions or after 48 h of fasting were significantly higher in *Ppara* ^∆KPT^ mice than in *Ppara* ^fl/fl^ controls (Figure 2C). Serum glucose levels after 48 h of fasting were significantly lower in *Ppara* ^∆KPT^ mice. However, serum insulin and glucagon levels were similar between the mouse genotypes at all assessment points (Figure S6A,B). Insulin and glucose sensitivity assessed through ITT and GTT exhibited similar results between the mouse groups (Figure S6C,D).

### 3.3 FAO, Ketogenesis, and Gluconeogenesis in the Kidney Were Insufficiently Accelerated With Fasting in *Ppara* ^∆KPT^ Mice

**FIGURE 3:**
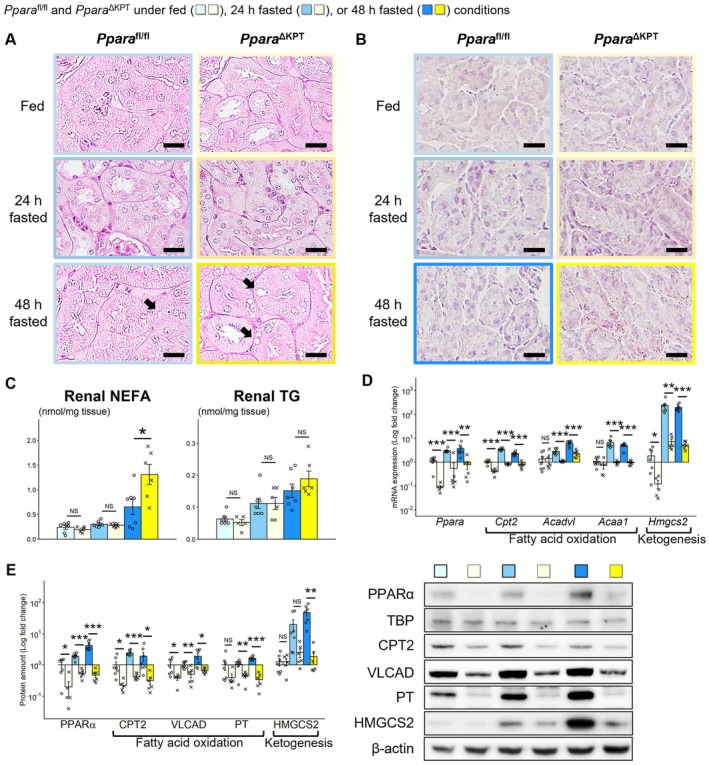
Renal lipid metabolism during fasting. (A) Light microscopic analysis with periodic acid‐Schiff staining. Microvacuoles in the proximal tubule are indicated with arrows. Scale bar = 25 μm. (B) Light microscopic analysis with Oil red O staining. (C) Renal content of nonesterified fatty acid (NEFA) and triglyceride (TG) was measured in a biochemical manner. (D) mRNA expression of fatty acid oxidation and ketogenesis‐related genes. (E) Protein expression about fatty acid oxidation and ketogenesis. A significant difference between the groups of *Ppara* ^∆KPT^ and *Ppara* ^fl/fl^ controls with the same fasting time is indicated with asterisks. *: *p* < 0.05. **: *p* < 0.01. ***: *p* < 0.001. No significant differences are indicated with NS.

**FIGURE 4:**
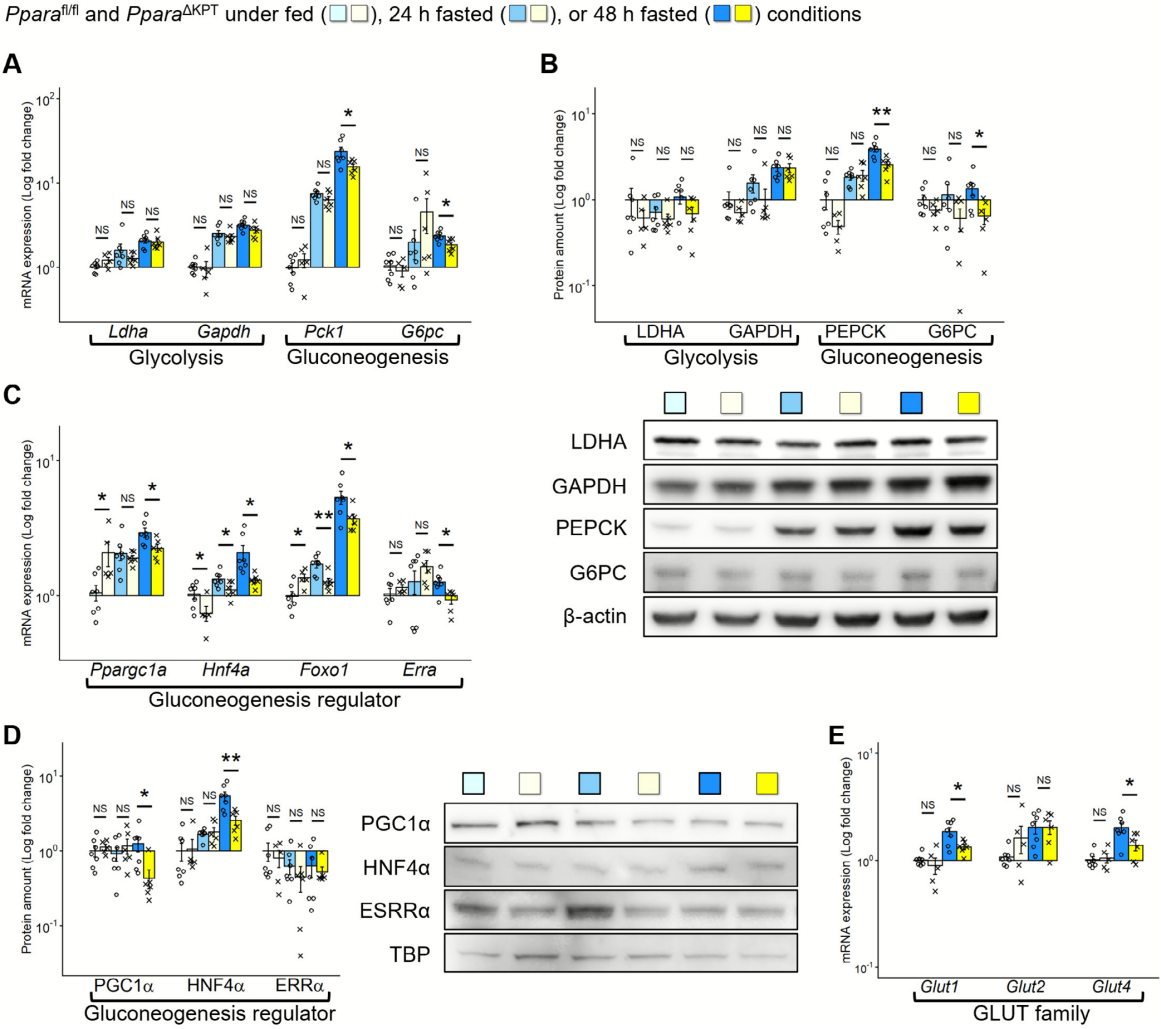
Renal glucose metabolism during fasting. (A) Renal mRNA expression of glycolysis and gluconeogenesis‐related genes. (B) Protein expression of glycolysis and gluconeogenesis‐related genes. (C) mRNA expression of gluconeogenesis‐related transcription factors. (D) Protein expression of gluconeogenesis‐related transcription factors. (E) mRNA expression of glucose transporters under fed or 48 h fasted conditions. A significant difference between the groups of *Ppara* ^∆KPT^ and *Ppara* ^fl/fl^ controls with the same fasting time is indicated with asterisks. *: *p* < 0.05. **: *p* < 0.01. ***: *p* < 0.001. No significant differences are indicated with NS.

*Ppara* ^∆KPT^ mice did not exhibit abnormal glomeruli or tubular injury, even after 48 h of fasting. However, many more microvacuoles were detected in the PT (PT‐vacuoles) of *Ppara* ^∆KPT^ mice after 48 h of fasting relative to *Ppara* ^fl/fl^ controls (Figure 3A). The amount of extracted whole lipids was higher in *Ppara* ^∆KPT^ mice after 48 h of fasting (Figure S7A). Oil red O staining and biochemical analysis also revealed that significantly more lipids accumulated in the kidneys of *Ppara* ^∆KPT^ mice after 48 h of fasting (Figure 3B,C). Measurement of mRNA and protein levels of FAO‐related genes, and ketogenesis‐related genes were defective in *Ppara* ^∆KPT^ mice and were significantly increased with fasting only in *Ppara* ^fl/fl^ controls but not in *Ppara* ^∆KPT^ mice (Figure 3D,E). Protein expression of RXRα, which forms a heterodimer with PPARα and regulates downstream gene trasncriptions [11, 21], exhibited similar levels between the groups both under fed and fasted conditions (Figure S7B). The *Ppard* and *Pparg* mRNA levels were similar between the groups after 48 h of fasting, whereas *Pparg* levels were higher in *Ppara* ^∆KPT^ mice under fed conditions (Figure S7C). Protein levels of p‐mTOR, a PPARα‐associated regulator for energy metabolism [22], were similar between the groups, both under fed and fasted conditions (Figure S7D). Although renal mRNAs of glycolysis‐related genes were similarly expressed between the mouse types, those of GNG‐related genes were significantly lower in *Ppara* ^∆KPT^ mice after 48 h of fasting than in *Ppara* ^fl/fl^ controls (Figure 4A,B). GNG‐related transcription factor [23] mRNAs were also expressed at lower levels in *Ppara* ^∆KPT^ mice after 48 h of fasting (Figure 4C,D). We assessed mRNA levels of glucose transporter family (GLUTs) and found that the *Glut1* and *Glut4* levels were significantly lower in *Ppara* ^∆KPT^ compared to *Ppara* ^fl/fl^ controls after 48 h of fasting (Figure 4E). mRNA expression of fibroblast growth factor 21 (*Fgf21*), encoded by a PPARα target gene possibly linking multiple organs‐metabolism [24], was similar between the mouse types at each assessment point (Figure S7E). To assess the potential association of PPARα in PT for systemic metabolism through inflammation pathways, we assessed mRNA levels of cytokine‐associated genes (*Il6* and *Tnfa*) in the kidneys of the mice. However, the expression was notably decreased with fasting and not significantly different between the mouse genotypes, both under fed and fasted conditions (Figure S7F). These results indicate that the kidneys of *Ppara* ^∆KPT^ mice had insufficient FAO, ketogenesis, and GNG ability, which may cause excessive lipid accumulation in the kidney and lower serum glucose levels during fasting.

### 3.4 Hepatic Fatty Acid Metabolism and Glucose Metabolism During Fasting Were More Activated in *Ppara* ^∆KPT^ Mice

**FIGURE 5:**
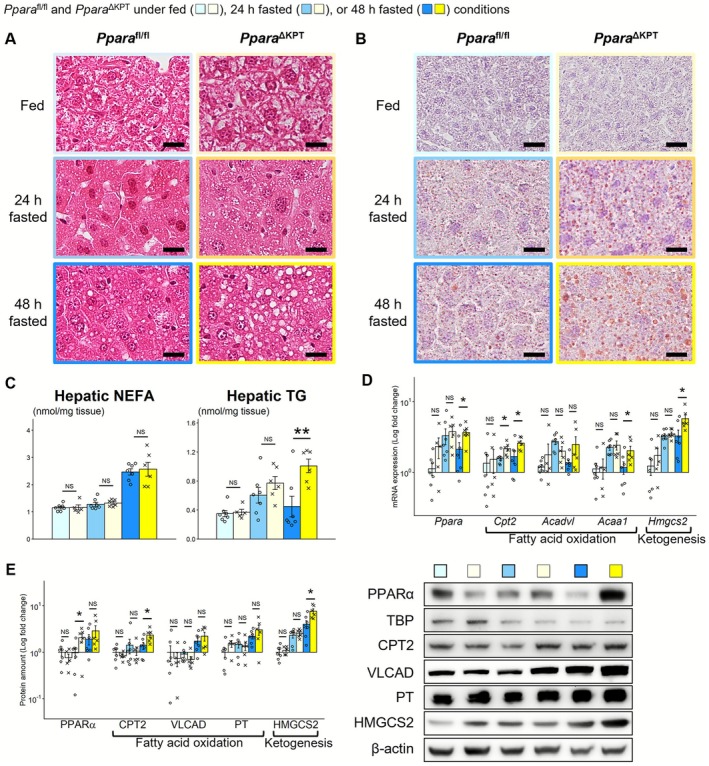
Hepatic fatty acid metabolism during fasting. (A) Light microscopic analysis of the liver with hematoxylin and eosin staining. Scale bar = 25 μm. (B) Light microscopic analysis of the liver with Oil Red O staining. (C) Hepatic content of nonesterified fatty acid (NEFA) and triglyceride (TG) measured by biochemical methods. (D) mRNA expression of fatty acid oxidation and ketogenesis‐related genes. (E) Protein expression of fatty acid oxidation and ketogenesis‐related genes. A significant difference between the groups of *Ppara* ^∆KPT^ and *Ppara* ^fl/fl^ controls with the same fasting time is indicated with asterisks. *: *p* < 0.05. **: *p* < 0.01. No significant differences are indicated with NS.

**FIGURE 6:**
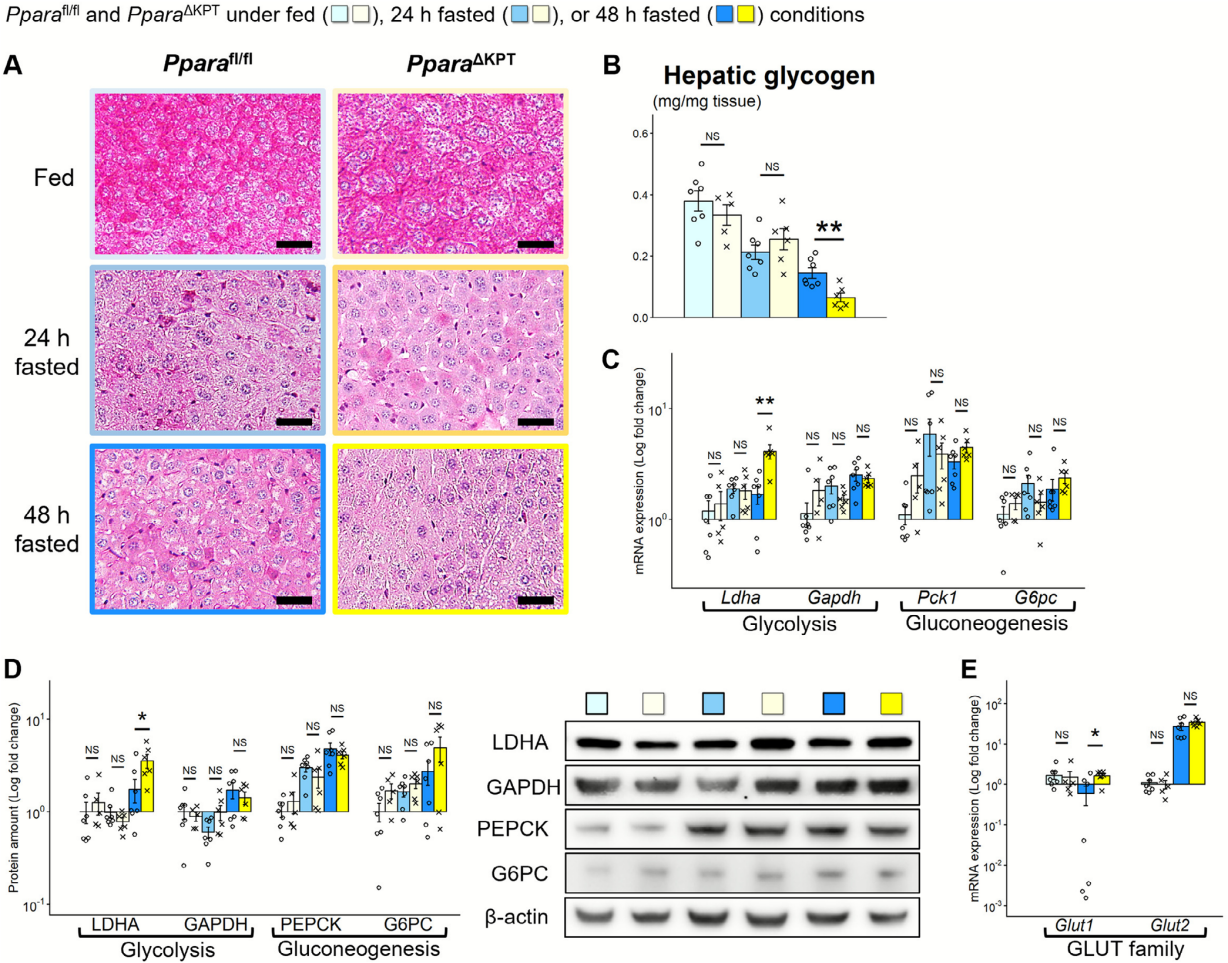
Hepatic glucose metabolism during fasting. (A) Light microscopic analysis with periodic acid‐Schiff staining for glycogen assessment. (B) The hepatic content of glycogen is measured in a biochemical manner. (C) mRNA expression of glycolysis and gluconeogenesis‐related genes. (D) Protein expression of glycolysis and gluconeogenesis‐related genes. (E) mRNA expression of glucose transporters underfed or 48 h fasted conditions. A significant difference between the groups of *Ppara* ^∆KPT^ and *Ppara* ^fl/fl^ controls with the same fasting time is indicated with asterisks. *: *p* < 0.05. **: *p* < 0.01. No significant differences are indicated with NS.

Light microscopy analysis and Oil red O staining revealed more extensive hepatic micro‐steatosis in *Ppara* ^∆KPT^ mice after 48 h of fasting compared to that in *Ppara* ^fl/fl^ controls, suggesting the presence of acute hepatic metabolic changes (Figure 5A,B). Although there was no significant difference in the amount of extracted whole lipids between the groups (Figure S8A), higher TG accumulation was observed in *Ppara* ^∆KPT^ mice after 48 h of fasting compared to that in *Ppara* ^fl/fl^ controls (Figure 5C). Although hepatic levels of PPARα, FAO, and ketogenesis‐related gene mRNAs and proteins were comparable under fed conditions between the mouse genotypes, they were significantly increased with fasting in *Ppara* ^∆KPT^ mice (Figure 5D,E). Histological analysis with PAS staining and biochemical measurements revealed that hepatic glycogen levels were significantly lower after 48 h of fasting in *Ppara* ^∆KPT^ mice (Figure 6A,B). Although GNG‐related genes were similarly expressed in the liver between the mouse genotypes, the expression of *Ldha* mRNA encoding an enzyme involved in glycolysis was more increased with fasting in *Ppara* ^∆KPT^ mice (Figure 6C,D). *Glut1* mRNA expression was significantly higher in *Ppara* ^∆KPT^ mice compared to *Ppara* ^fl/fl^ controls after 48 h fasting (Figure 6E). mRNA levels of *Fgf21* and cytokine‐associated genes were similar between the mouse genotypes at each assessment point (Figure S8B,C). These results suggest that hepatic lipid and glucose metabolism during fasting is further accelerated in *Ppara* ^∆KPT^ mice to compensate for impaired lipid and glucose metabolism in the kidney.

### 3.5 Metabolism in White Adipose Tissue, Skeletal Muscle, and Heart During Fasting Was Influenced by Loss of PPARα in PT

**FIGURE 7:**
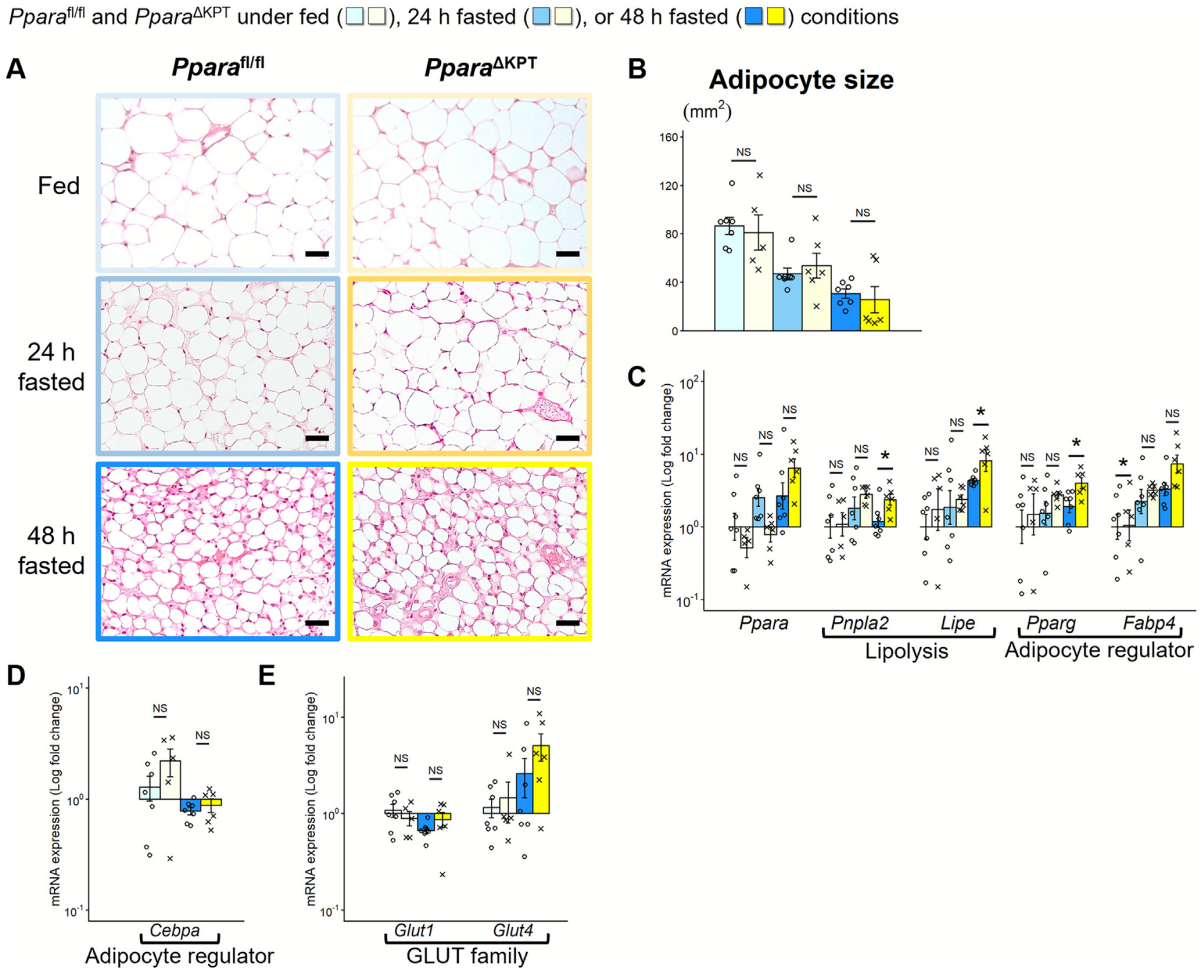
Lipid metabolism in white adipose tissue during fasting. (A) Light microscopic analysis with hematoxylin and eosin staining of epididymal white adipose tissue. Scale bar = 50 μm. (B) The average size of an adipocyte. (C) mRNA expression of PPARα, lipolysis‐related genes, and regulator genes of adipocyte metabolism. (D and E) mRNA expression of *Cebpa* and glucose transporters under fed or 48 h fasted conditions. A significant difference between the groups of *Ppara* ^∆KPT^ and *Ppara* ^fl/fl^ controls with the same fasting time is indicated with asterisks. *: *p* < 0.05. No significant differences are indicated with NS.

Adipocyte size in eWAT was decreased by fasting in both mouse genotypes (Figure 7A,B). However, mRNA levels of *Ppara* and lipolysis‐related genes, and regulator genes of adipocyte metabolism at 48 h of fasting were accelerated in *Ppara* ^∆KPT^ mice than in *Ppara* ^fl/fl^ controls (Figure 7C). mRNA expression of *Cebpa*, another regulator gene for adipocyte differentiation [25], and GLUTs was similar between the groups, both under fed and fasting conditions (Figure 7D,E). In the GM, *Ppara* mRNA expression was significantly higher in *Ppara* ^∆KPT^ mice than in fasted *Ppara* ^fl/fl^ controls after 48 h of fasting (Figure S9A). However, the mRNA expression of FAO and glycolysis genes was similar between the mouse genotypes (Figure S9A,B). Several mRNAs related to muscle catabolism were significantly more expressed in *Ppara* ^∆KPT^ mice after 48 h of fasting than in *Ppara* ^fl/fl^ controls (Figure S9C). mRNA levels of GLUTs were similar between the groups (Figure S9D). Cardiac mRNA expression of *Ppara* and glycolysis‐related genes was similar between the mouse types at all assessment points (Figure S10A,B). However, CPT2, encoded by a key gene involved in FAO, was highly expressed in *Ppara* ^∆KPT^ mice after 48 h of fasting relative to *Ppara* ^fl/fl^ controls.

## 4 Discussion

PT produces ATP, mainly by FAO, to maintain its functions [7]. In the present study, renal transcription of FAO‐related genes was diminished in *Ppara* ^∆KPT^ mice, and it did not increase even during fasting, which activates PPARα signaling and FAO [9]. These results indicate that PPARα in PT is indispensable in activating renal FAO and also in the homeostasis of renal energy metabolism. Earlier studies reported that fasted global *Ppara* ^−/−^ mice exhibited deficient FAO and insufficient ATP production in their kidneys [13]. Although global *Ppara* ^−/−^ mice developed severe albuminuria during fasting, this phenotype was not observed in fasted *Ppara* ^∆KPT^ mice in the present study. Since PPARα expression in the liver and GM was increased by fasting in *Ppara* ^∆KPT^ mice compared to *Ppara* ^fl/fl^ controls, the supply of energy substrates by extra‐renal PPARα activation might rescue the renal metabolism of *Ppara* ^∆KPT^, maintain urine reabsorption ability, and prevent the development of albuminuria. Light microscopic analysis revealed that *Ppara* ^∆KPT^ mice had many PT‐vacuoles after 48 h of fasting. PT‐vacuoles are found in various experimental models and may result from the accumulation of a variety of substances [26]. Owing to increased lipid accumulation in the kidney of fasted *Ppara* ^∆KPT^, the vacuoles may contain an excessive amount of lipid droplets. Others reported that PT‐vacuoles found in an obese mouse model are lysosomes phagocytosing lipid droplets [26]. Fasted global *Ppara* ^−/−^ mice exhibited numerous PT‐vacuoles, which are malformed lysosomes likely resulting from deficient energy flux induced by the loss of kidney PPARα [13]. As PPARα and PPARα‐associated nuclear receptors, including RXRα and PGC1α, directly regulate lysosomal biogenesis [27], vacuoles in the PT of fasted *Ppara* ^∆KPT^ mice might develop through malformed lysosome biogenesis. Although it remains unclear whether the vacuoles observed in fasted *Ppara* ^∆KPT^ mice are lipid droplets, lysosomes, other substances, or a combination of those, previous studies and the present results suggest that PPARα in PT is involved in lysosomal metabolism. While the importance of PPARα in various types of renal metabolism was reported in previous studies, these studies were based on experiments using global *Ppara* ^−/−^ mice or systemic PPARα agonists [13, 14, 15, 16]. Recently, genome‐wide transcriptomic analysis revealed decreased expression of PPARα and FAO‐related genes in the kidneys of patients with chronic kidney disease (CKD), underscoring the importance of maintaining renal FAO ability [28]. With the current results, we speculate that PPARα in PT has a renoprotective role, and have planned further experiments using kidney disease models on *Ppara* ^∆KPT^ to clarify this issue.

In the present study, *Ppara* ^∆KPT^ mice exhibited significantly lower serum glucose levels than *Ppara* ^fl/fl^ controls after 48 h of fasting. After about 12 h from the initiation of fasting, the maintenance of serum glucose level gradually shifts from glycogenolysis to GNG [29]. Although the glucose production ratio of hepatic and renal GNG is initially at 7:3, the contribution of the kidney increases with fasting duration and ultimately reaches a level equivalent to that of the liver [30]. Given that the mice with 48 h of fasting in the present study exhibited exhaustion of hepatic glycogen and a significant decrease in BW, those mice may be under a condition requiring huge renal GNG. Indeed, renal transcription of GNG‐related genes was increased less in *Ppara* ^∆KPT^ mice after 48 h of fasting, suggesting that insufficient renal GNG caused lower serum glucose levels in fasted *Ppara* ^∆KPT^ mice. PPARα controls not only lipid metabolism but also influences glucose metabolism, and fasted global *Ppara* ^−/−^ mice are widely reported to develop severe hypoglycemia [9, 12]. Furthermore, fasted global *Ppara* ^−/−^ mice reportedly exhibit more severe hypoglycemia than fasted liver‐specific *Ppara*‐null (*Ppara* ^∆HEP^) mice, suggesting the contribution of extra‐hepatic PPARα to systemic glucose metabolism [31]. Although it is controversial how PPARα contributes to glucose metabolism and maintenance of serum glucose levels, several studies have associated PPARα with GNG [32]. Others reported the insufficient transcription of GNG‐related genes in the liver of fasted global *Ppara* ^−/−^ mice [33]. Chromatin immunoprecipitation analysis with rat hepatocytes revealed that PPARα is recruited to the promoter region of glucose 6‐phosphatase (*G6pc*), a key gene for GNG, together with several transcription factors, including hepatocyte nuclear factor 4‐alpha (HNF4α) [34]. Although some studies have reported results that do not support a direct association of PPARα with GNG [32, 35], the present study demonstrated that 48 h of fasting *Ppara* ^∆KPT^ mice exhibited lower expression of G6PC and GNG regulators, including HNF4α and peroxisome proliferator‐activated receptor gamma coactivator 1‐alpha. These results suggest that PPARα in PT controls renal GNG with multiple transcription factors and contributes to the homeostasis of systemic glucose metabolism. It has previously been reported that hepatic ketogenesis and increased serum ketone levels activate renal GNG during fasting [36]. In that study, renal GNG was activated by ketone injection even in global *Ppara* ^−/−^ mice, suggesting that serum ketone activates renal GNG without PPARα in the kidney. In the present study, however, fasted *Ppara* ^∆KPT^ mice exhibited decreased renal GNG despite more activated hepatic ketogenesis and higher serum ketone levels compared to fasted *Ppara* ^fl/fl^ controls. Therefore, serum ketone and renal PPARα may independently regulate renal GNG, and reduced renal GNG in fasted *Ppara* ^∆KPT^ mice in the present study may be partially alleviated by hepatic ketogenesis and higher serum ketone levels. Recently, several studies have suggested the possible renoprotective role of GNG in PT [23, 37, 38]. Further assessment of the association between PPARα and GNG could help develop novel treatment strategies for kidney diseases. Another notable result of this study is that mRNA expression of *Glut1* and *Glut4* was decreased in fasted *Ppara* ^∆KPT^ mice compared to *Ppara* ^fl/fl^ controls. GLUTs are transmembrane proteins that regulate the entry of extracellular glucose into cells and its exit from the cells [39]. Although previous studies have focused on the role of *Glut1* and *Glut4* in the glomerulus, thick ascending limb, or vascular smooth muscle [39], several studies have addressed the potential effect of those GLUTs in PT for renal and systemic glucose metabolism [40, 41]. Additionally, other studies reported that PPARα binds to GATA6, a nuclear receptor, leading to the direct activation of GLUT4 transcription and improving glucose consumption in myoblasts [42]. Therefore, PPARα deletion in PT possibly affects glucose metabolism through the regulation of GLUTs in the kidney.

**FIGURE 8:**
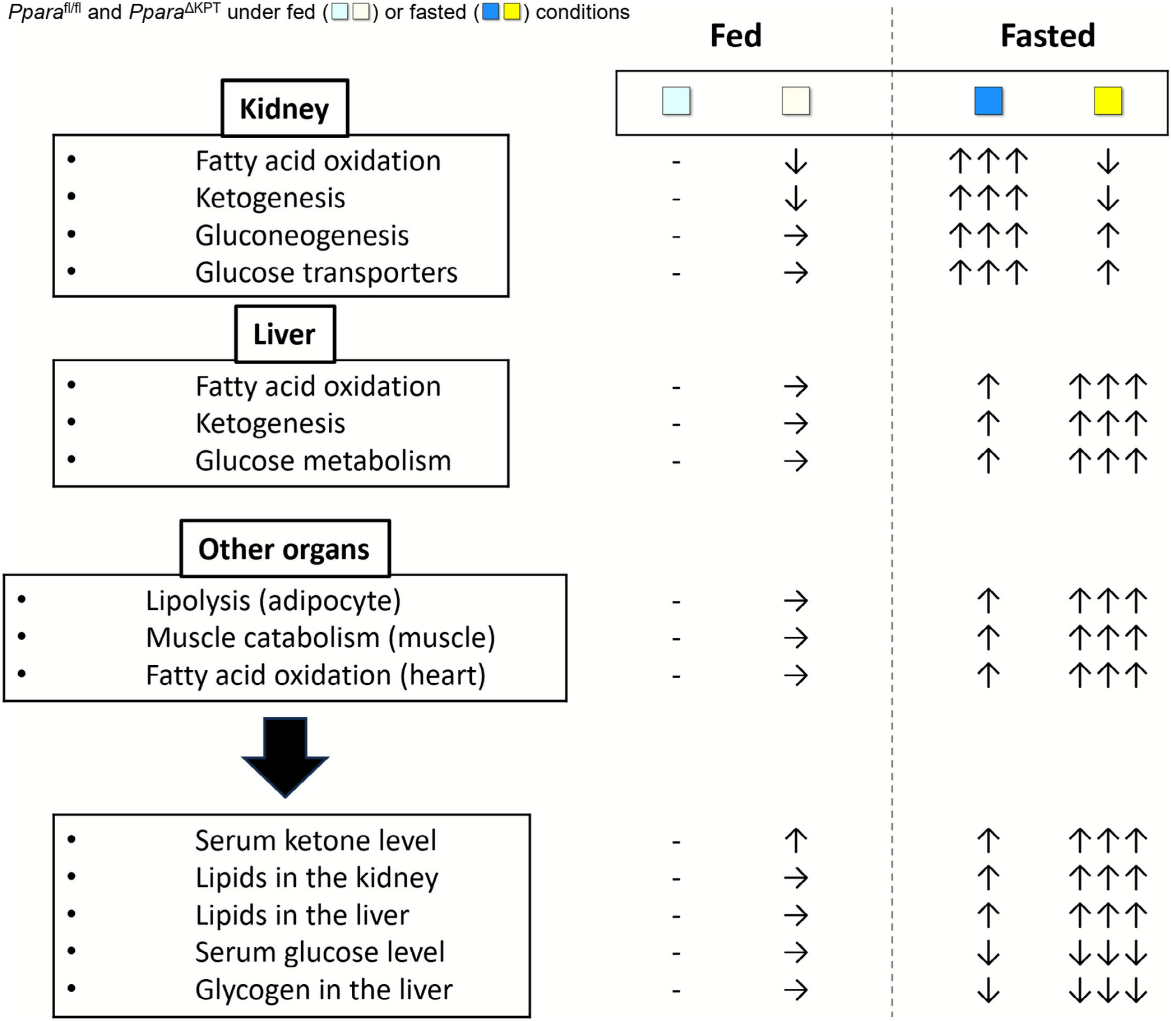
Summary of metabolic changes in fasted *Ppara* ^∆KPT^ mice. Metabolic changes in the examined organs of *Ppara* ^∆KPT^ mice and *Ppara* ^fl/fl^ controls underfed and fasted conditions.

The present study also revealed that disruption of the *Ppara* gene in PT affects metabolism in extra‐renal organs, including the liver, eWAT, GM, and heart (Figure 8). Others reported that fasted *Ppara* ^∆HEP^ mice showed mild hypoglycemia, hypoketonemia, hypothermia, and accumulation of hepatic TG compared to that shown by fasted global *Ppara* ^−/−^ mice [31]. These previous results and our findings indicate that PPARα in PT contributes to the homeostasis of systemic energy metabolism during fasting. However, its mechanisms involved are unknown. Although PPARα in PT may contribute to systemic metabolism through renal GNG and maintenance of serum glucose levels, serum levels of insulin and glucagon—major hormones in the starvation response manipulated by blood glucose level [43]—were similar in the different mouse types after 48 h of fasting in the present study. Additionally, sensitivity for insulin and glucagon assessed by ITT and GTT was also similar between the mouse genotypes. Several studies have proposed that FGF21, a soluble molecule regulated by PPARα, plays a key role in kidney‐associated systemic lipid and glucose metabolism [24, 44]. However, we did not find any difference in renal and hepatic *Fgf21* gene expression between *Ppara* ^∆KPT^ mice and *Ppara* ^fl/fl^ controls. We also assessed the potential hypothesis that the presence of PPARα in PT influences systemic metabolism through inflammatory pathways. However, the expression of cytokine‐associated genes in the kidney and liver exhibited similarities between the mouse genotypes and decreased with fasting. To our knowledge, no study has assessed the effect of PPARα in PT on extra‐renal metabolism; therefore, further research is needed. Patients with CKD have decreased renal expression of PPARα [28] and frequently suffer from various systemic disorders, including dyslipidemia, hypoglycemia, malnutrition, frailty, and sarcopenia [45, 46, 47, 48]. Since *Ppara* ^∆KPT^ mice, during fasting, showed hepatic micro‐steatosis with further lipid accumulation, lower serum glucose levels, and increased transcription of muscle catabolism‐related genes than those in *Ppara* ^fl/fl^ controls, impaired activation of PPARα in PT in patients with CKD could cause the development of such systemic complications. Furthermore, patients with CKD are known to have a higher risk of hypoglycemia than cases with normal kidney function, and the risk of various adverse events increases during fasting, such as Ramadan, depending on kidney dysfunction [49]. These clinical findings may relate to the inactivity of PPARα in PT in patients with CKD. Additionally, fasting has a physiological commonality with obesity and diabetes in that both conditions require organs, including the liver, to metabolize fatty acids [50]. Therefore, PPARα in PT is expected to play a crucial role in systemic lipid metabolism against obesity and diabetes. Further studies will lead to a better understanding of the role of the kidney in systemic metabolism and the mechanism of systemic complications associated with CKD.

This study had some limitations. First, the present study was conducted with a relatively mild single 48 h of fasting, due to ethical restrictions based on animal welfare. To detect significant phenotypic changes, such as poorer survival ratio, further BW loss, and functional abnormalities of tubule and external organs, including the liver, adipocyte, muscle, and heart, more studies with more severe fasting protocols, such as multiple and intermittent, may be necessary. Second, our analyses were focused mainly on fatty acid, ketone, and glucose metabolism, which were predicted to be altered from past study findings using global *Ppara* ^−/−^ mice. To investigate other gene expression changes comprehensively, whole transcriptomics in PT or in other organs would be required. Finally, the association between PPARα in PT and renal GNG has not been sufficiently evaluated. Assessment of GNG is an elusive and challenging issue, and analysis using isotope tracing is ideally recommended for the precise measurement of GNG flux [51]. Although the GNG of *Ppara* ^∆KPT^ mice was assessed as the transcription level of GNG‐related genes in this study, assessment with an established measurement method is preferable to strengthen our results and hypothesis.

In conclusion, we revealed that PPARα in PT plays a pivotal role in renal FAO, ketogenesis, and GNG, contributing to the homeostasis of renal and systemic energy metabolisms. Our results may lead to a better understanding of the systemic disorders in patients with CKD who have impaired renal PPARα activation.

## Author Contributions

D. Aomura, Y. Yamada, and Y. Kamijo conceptualized the project. D. Aomura conducted the experiments and analyses with T. Nimura and K. Yamaka. D. Aomura wrote and reviewed the manuscript and was supported by M. Harada and T. Nakajima. T. Kimura, K. Hashimoto, and H. Kanno revised the article for intellectual content. M. Yanagita, F. Gonzalez, and N. Tanaka provided critical materials, reviewed the experimental design, and edited the manuscript. Y. Kamijo conceived and designed the study, edited the manuscript, and led the research team.

## Funding

This study was supported by a Grant‐in‐Aid for Scientific Research (KAKENHI) from Japan (22 K08306) and the National Cancer Institute Intramural Research Program (1ZIABC005708‐32).

## Conflicts of Interest

M.Y. has received research grants from Mitsubishi Tanabe Pharma and Boehringer Ingelheim.

## Supporting information


**Data S1:** Supporting Information S1.

**Data S2:** DataSet.

## Data Availability

The sample data that support the findings of this study are available in the Supporting Information.
