# Supplementary material for: Proximal Tubule‐Specific Genetic Deficiency of PPARα Worsens Systemic Lipid and Glucose Metabolism During Fasting
Source: FASEB J. 2025 Dec 29;40(1):e71333. doi: 10.1096/fj.202502975R (PMC12747219; doi:10.1096/fj.202502975R)
Supplement: Supplementary file 1 — Data S1: Supporting Information S1. [file FSB2-40-e71333-s002.docx]

**Supplementary Tables**

**Supplementary Table 1:** Primers for genotyping

| For floxed *Ppara* | Primer sequence (5′′–3′′) |
| --- | --- |
| 5′ primer | CAAGGCCATGTCTAATCATCCTGG |
| 3′ wild type-specific primer | TCTCATGGATTCAATTGACTGACTGG |
| 3′ floxed *Ppara*-specific primer | CAACGGGTTCTTCTGTTAGTCC |
| For *Ndrg1*-CreER^T2^ | Primer sequence (5′–3′) |
| 5′ primer | GTCTGGACTAGGTGTGAACCTGC |
| 3′ wild type-specific primer | CTCAAGAGTGTCCATGCCAATATG |
| 3′ *Ndrg1*-CreER^T2^-specific primer | TGGTGTACGGTCAGTAAATTGG |

**Supplementary Table 2:** Primers for real-time polymerase chain reaction

| Gene |  | Primer sequence (5′–3′) | GenBank accession number |
| --- | --- | --- | --- |
| *Ppara* | Forward | CCTCAGGGTACCACTACGGAGT | NM_011144 |
|  | Reverse | GCCGAATAGTTCGCCGAA |  |
| *Tbp* | Forward | ATAAGAGAGCCACGGACAACTG | NM_013684 |
|  | Reverse | CTGCTAGTCTGGATTGTTCTTCAC |  |
| *Cpt2* | Forward | ATCGTACCCACCATGCACTAC | NM_009949 |
|  | Reverse | CTGTCATTCAAGAGAGGCTTCTG |  |
| *Acadvl* | Forward | GCGTGTGCTCCGAGATATTC | NM_017366 |
|  | Reverse | CCAGTGAGTTCCTTTCCTTTG |  |
| *Acaa1* | Forward | TCTACGGTCAACAGACAGTGTTCA | NM_146230 |
|  | Reverse | GGCCATGCCAATGTCATAAGA |  |
| *Hmgcs2* | Forward | GATACCACCAACGCCTGTTATG | NM_008256 |
|  | Reverse | CATAGCGACCATCCCAGTAGC |  |
| *Actb* | Forward | GCCTTCCTTCTTGGGTATGG | NM_007393 |
|  | Reverse | GTGTTGGCATAGAGGTCTTTACG |  |
| *Fgf21* | Forward | CCTCTAGGTTTCTTTGCCAACAG | NM_020013 |
|  | Reverse | AAGCTGCAGGCCTCAGGAT |  |
| *Ldha* | Forward | CAAAGACTACTGTGTAACTGCGA | NM_001136069 |
|  | Reverse | TGGACTGTACTTGACAATGTTGG |  |
| *Gapdh* | Forward | TGCACCACCAACTGCTTAG | NM_001289726 |
|  | Reverse | GGATGCAGGGATGATGTTCTG |  |
| *Pck1* | Forward | GTGTCATCCGCAAGCTGAAGA | NM_011044 |
|  | Reverse | CTTTCGATCCTGGCCACATCT |  |
| *G6pc* | Forward | TTACCAAGACTCCCAGGACTG | NM_008061 |
|  | Reverse | GAGCTGTTGCTGTAGTAGTCG |  |
| *Ppargc1a* | Forward | GACTCAGTGTCACCACCGAAATC | NM_001402987 |
|  | Reverse | GACCTGTGTCGAGAAAAGGATCTT |  |
| *Hnf4a* | Forward | CAAGAGGTCCATGGTGTTTAAGG | NM_001312906 |
|  | Reverse | GGATGGACACACGGCTCATC |  |
| *Foxo1* | Forward | ACATTTCGTCCTCGAACCAGCTCA | NM_019739 |
|  | Reverse | ATTTCAGACAGACTGGGCAGCGTA |  |
| *Esrra* | Forward | AGGAAGACAGCCCCAGTGAAC | NM_001413228 |
|  | Reverse | GACCACTATCTCTCGATCAAAAAGGT |  |
| *Pnpla2* | Forward | ACGGGAAGAACAAGATTGGAAG | NM_001163689 |
|  | Reverse | CGTTCCCTCAAACATAGGGC |  |
| *Lipe* | Forward | GAGCGCTGGAGGAGTGTTTT | NM_001039507 |
|  | Reverse | TGATGCAGAGATTCCCACCTG |  |
| *Fabp4* | Forward | TTTCCTTCAAACTGGGCGTG | NM_001409513 |
|  | Reverse | AGGGTTATGATGCTCTTCACCTTC |  |
| *Map1lc3b* | Forward | CAAGGGAAGTGATCGTCGCC | NM_001364358 |
|  | Reverse | TCGCTCTATAATCACTGGGATC |  |
| *Sqstm1* | Forward | GAGGCACCCCGAAACATGG | NM_001290769 |
|  | Reverse | ACTTATAGCGAGTTCCCACCA |  |
| *Murf1* | Forward | ACCTGCTGGTGGAAAACATC | NM_001039048 |
|  | Reverse | CTTCGTGTTCCTTGCACATC |  |
| *Atrogin1* | Forward | AGGAGCGCCATGGATACTGT | NM_026346 |
|  | Reverse | GAAGTTCTTTTGGGCGATGC |  |
| *Ppard* | Forward | CCGCATGAAGCTCGAGTATGA | NM_001411509 |
|  | Reverse | TCCAAAGCGGATAGCGTTGT |  |
| *Pparg* | Forward | TTCCACTATGGAGTTCATGCTTGT | NM_001127330 |
|  | Reverse | TCCGGCAGTTAAGATCACACCTA |  |
| *Glut1* | Forward | TGTCGGGTATCAATGCTGTGT | NM_011400 |
|  | Reverse | GATACCGGAGCCGATGGTG |  |
| *Glut2* | Forward | GAAGGAACTCAGTACAGCAGTG | NM_031197 |
|  | Reverse | TCATCCACATTCAGTACAGGAC |  |
| *Glut4* | Forward | AAAAGTGCCTGAAACCAGAG | NM_009204 |
|  | Reverse | TCACCTCCTGCTCTAAAAGG |  |
| *Il6* | Forward | TCTCTGCAAGAGACTTCCATCC | NM_031168 |
|  | Reverse | ACAGGTCTGTTGGGAGTGGT |  |
| *Tnfa* | Forward | CGGAGTCCGGGCAGGT | NM_013693 |
|  | Reverse | GCTGGGTAGAGAATGGATGAACA |  |
| *Cebpa* | Forward | CAAGAACAGCAACGAGTACCG | NM_001287514 |
|  | Reverse | GTCACTGGTCAACTCCAGCAC |  |

**Supplementary Table 3:** Primary antibodies for immunoblotting

| Targeted protein | Catalogue number (Manufacturer) |
| --- | --- |
| PPARα | #sc9000 (Santa Cruz Biotechnology, CA, USA) |
| TBP | #ab63766 (Abcam, Cambridge, MA, USA) |
| CPT2 | Miyazawa S, et al.* |
| PT | Miyazawa S, et al.** |
| HMGCS2 | #ab137043 (Abcam, Cambridge, MA, USA) |
| β-Actin | #ab8227 (Abcam, Cambridge, MA, USA) |
| PEPCK | #ab70358 (Abcam, Cambridge, MA, USA) |
| G6PC | #22169-1-AP, (ProteinTech, IL, USA) |
| LDHA | #ab52488 (Abcam, Cambridge, MA, USA) |
| GAPDH | #ab9485 (Abcam, Cambridge, MA, USA) |
| PGC1α | #ab3242 (Abcam, Cambridge, MA, USA) |
| HNF4α | #sc8987 (Santa Cruz Biotechnology, CA, USA) |
| ESRRα | #sc32971 (Santa Cruz Biotechnology, CA, USA) |
| RXRα | #sc553 (Santa Cruz Biotechnology, CA, USA) |
| p-mTOR (S2448) | #ab109268 (Abcam, Cambridge, MA, USA) |
| Albumin | #A90-135 (Bethyl Laboratories, AL, USA) |

Abbreviations: PPARα, peroxisome proliferator activated receptor alpha; TBP, TATA-binding protein; CPT2, carnitine palmitoyl-transferase 2; VLCAD, very long-chain acyl-CoA dehydrogenase; PT, peroxisomal 3-ketoacyl-CoA thiolase; HMGCS2, 3-Hydroxy-3-Methylglutaryl-CoA Synthase 2; PEPCK, phosphoenolpyruvate carboxykinase; G6PC, glucose 6-phosphatase; LDHA, lactate dehydrogenase; GAPDH, glyceraldehyde 3-phosphate dehydrogenase; PGC1α, peroxisome proliferator-activated receptor gamma coactivator 1-alpha: HNF4α, hepatocyte nuclear factor 4-alpha; ESRRα, estrogen-related receptor-alpha; RXRα, retinoid x receptor alpha; p-mTOR, phospho-mammalian target of rapamycin. Details of antibodies for CPT2 and PT were described in ^*^Miyazawa S, et al. Purification and properties of carnitine octanoyltransferase and carnitine palmitoyltransferase from rat liver*.* *J Biochem*. 1983;94:529–542 and ^**^Miyazawa S, et al. T. The presence of a new 3-oxoacyl-CoA thiolase in rat liver peroxisomes*.* *Eur J Biochem*. 1980;103:589–596, respectively.

**
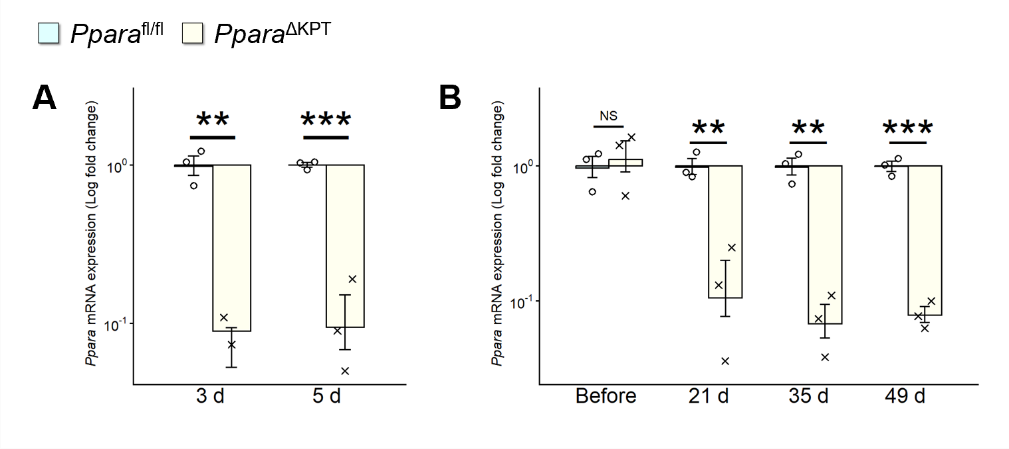
**

**Supplementary Figure 1. Renal PPARα deletion of *Ppara*^∆KPT^ mice with different tamoxifen-injection protocols**

mRNA expression of PPARα in the kidney of *Ppara*^∆KPT^ mice and *Ppara*^fl/fl^ controls with various tamoxifen-treatment protocols. (A) Mice were sacrificed on day 35 after tamoxifen (TM) injection for three or five days. (B) Mice were sacrificed before or on day 21, 35, or 49 days after TM injection for three days. A significant difference between *Ppara*^∆KPT^ mice and *Ppara*^fl/fl^ controls with the same protocol is indicated with asterisks. **: *p* < 0.01. ***: *p* < 0.001. No significant differences are indicated with NS.

**
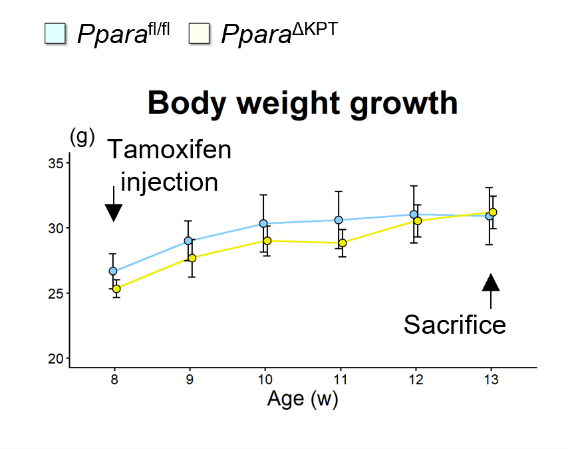
**

**Supplementary Figure 2.** **Body weight growth**

Body weight growth of *Ppara*^∆KPT^ mice and *Ppara*^fl/fl^ controls.

**
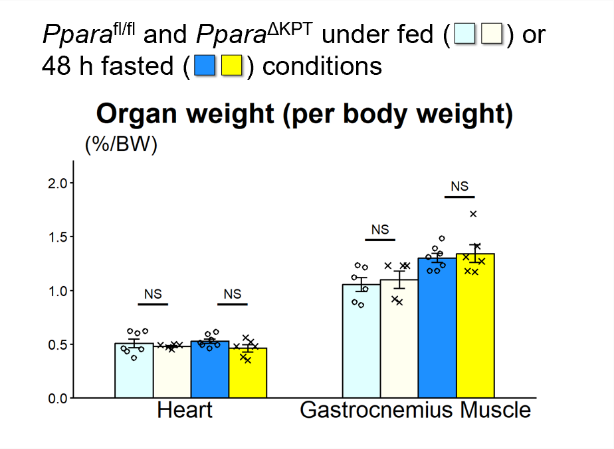
**

**Supplementary Figure 3. Decrease in heart and muscle weight during fasting**

Percentage of heart and gastrocnemius muscle weight per body weight before and after 48 h of fasting. No significant differences between the mouse genotypes are indicated with NS.

**
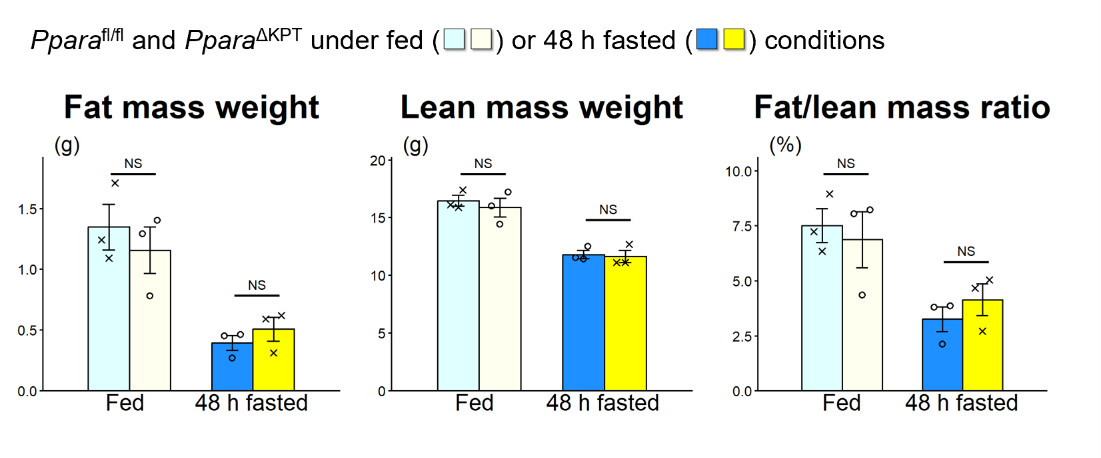
**

**Supplementary Figure 4. Body composition assessment by computed tomography imaging.**

Fat mass weight, lean mass weight, and the ratios in *Ppara*^∆KPT^ mice and *Ppara*^fl/fl^ controls at fed condition or after 48 h of fasting. Parameters were estimated from abdominal computed tomography images. No significant differences are indicated with NS.

**
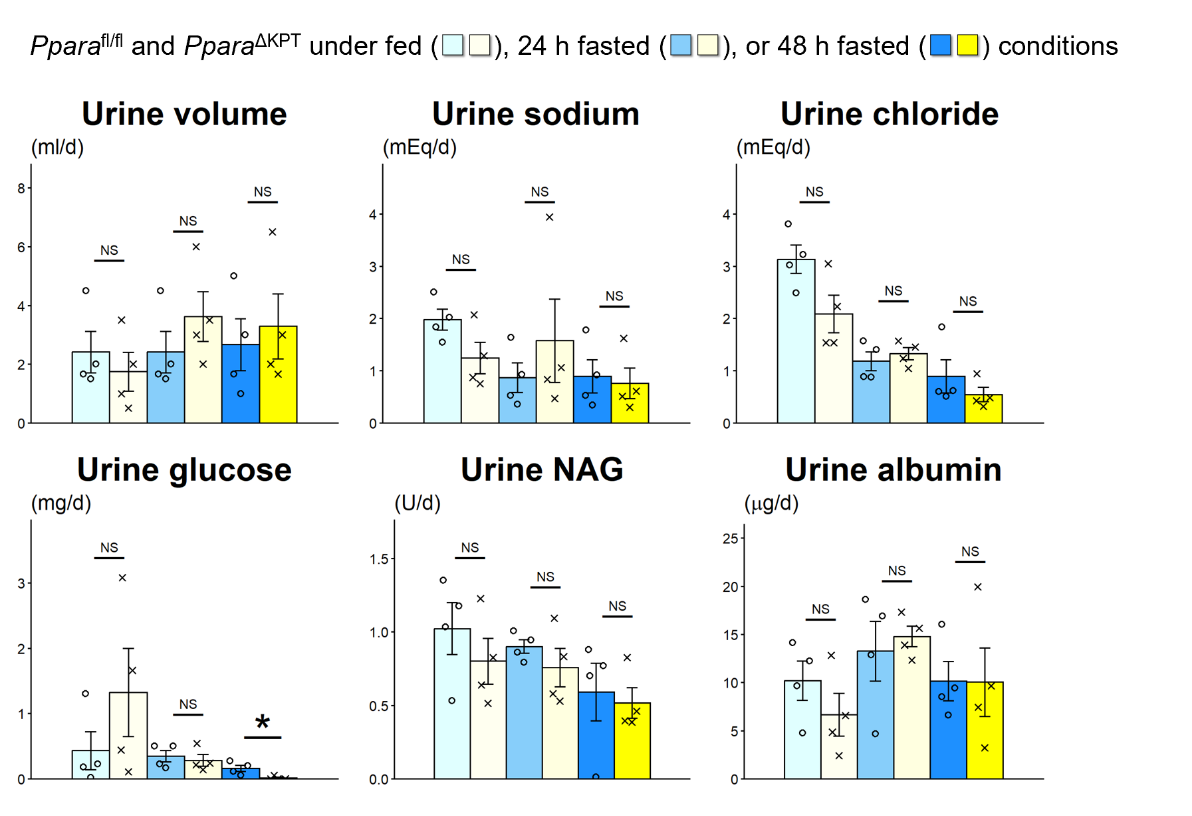
**

**Supplementary Figure 5. Urine analysis**

Urine analysis results in *Ppara*^∆KPT^ mice and *Ppara*^fl/fl^ controls during fasting. A significant difference between the groups of *Ppara*^∆KPT^ and *Ppara*^fl/fl^ controls with the same fasting time is indicated with asterisks. *: *p* < 0.05. No significant differences are indicated with NS.

**
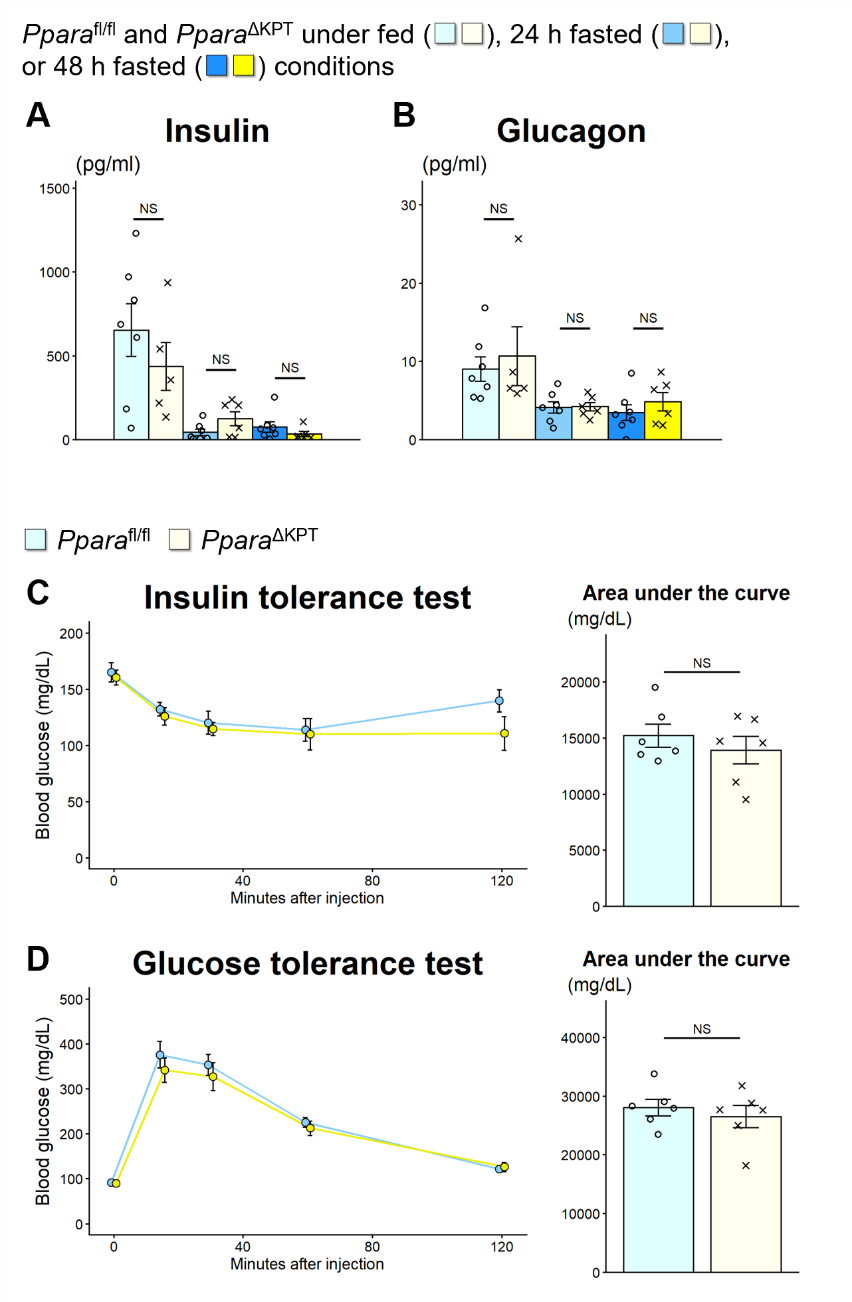
**

**Supplementary Figure 6. Insulin and glucose sensitivity**

(A and B) Serum insulin and glucagon levels in *Ppara*^∆KPT^ mice and *Ppara*^fl/fl^ controls during fasting. (C and D) Other *Ppara*^∆KPT^ mice and *Ppara*^fl/fl^ controls (n = 6 per group) were fasted for 16 h before intraperitoneal injection of insulin (0.5 units/kg body weight) or glucose (10 mg/kg body weight). Serum glucose levels were measured at 0, 15, 30, 60, and 120 min after injection. The area under the curve of serum glucose levels was compared between the mouse genotypes. No significant differences are indicated with NS.

**
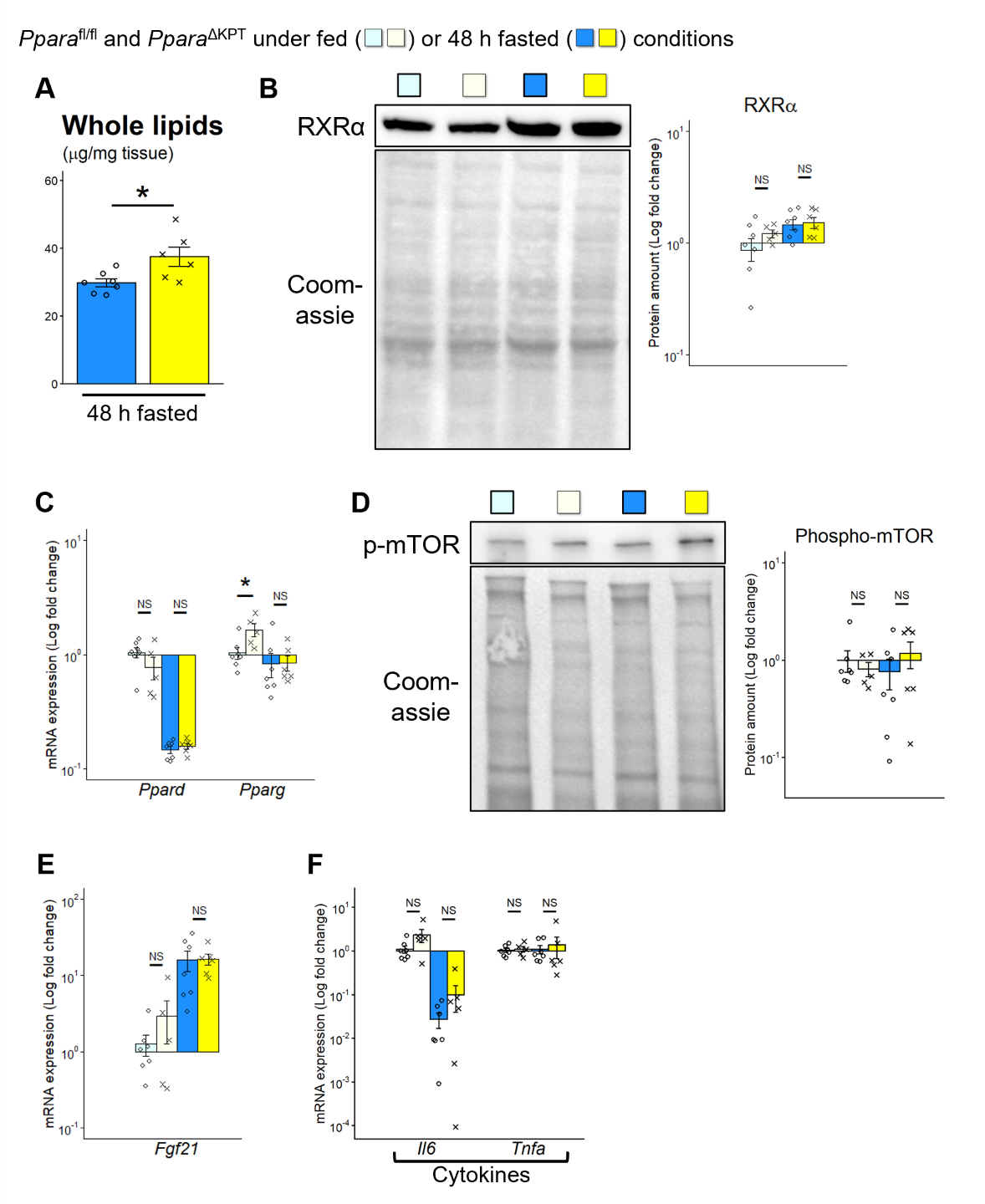
**

**Supplementary Figure 7. Changes in the kidney during fasting.**

(A) The amount of whole lipids extracted from the kidney of *Ppara*^∆KPT^ mice and *Ppara*^fl/fl^ controls after 48 h of fasting. (B-F) Protein or mRNA expression of RXRα, PPARs, phospho-mTOR, *Fgf21*, and cytokine-associated genes in the kidney during fasting. A significant difference between *Ppara*^∆KPT^ mice and *Ppara*^fl/fl^ controls with the same fasting time is indicated with asterisks. Protein expression was standardized with the total protein amount assessed through Coomassie staining. *: *p* < 0.05. No significant differences are indicated with NS.

**
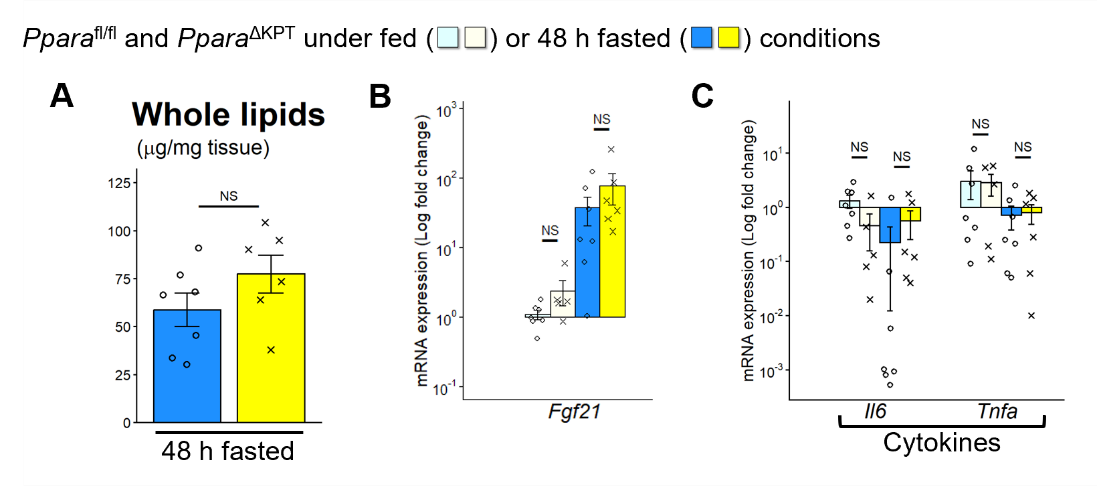
**

**Supplementary Figure 8. Changes in the liver during fasting**

(A) The amount of whole lipids extracted from the liver of *Ppara*^∆KPT^ mice and *Ppara*^fl/fl^ controls after 48 h of fasting. (B and C) mRNA expression of *Fgf21* and cytokine-associated genes in the liver during fasting. Difference between *Ppara*^∆KPT^ mice and *Ppara*^fl/fl^ controls with the same fasting time was assessed. No significant differences are indicated with NS.

**
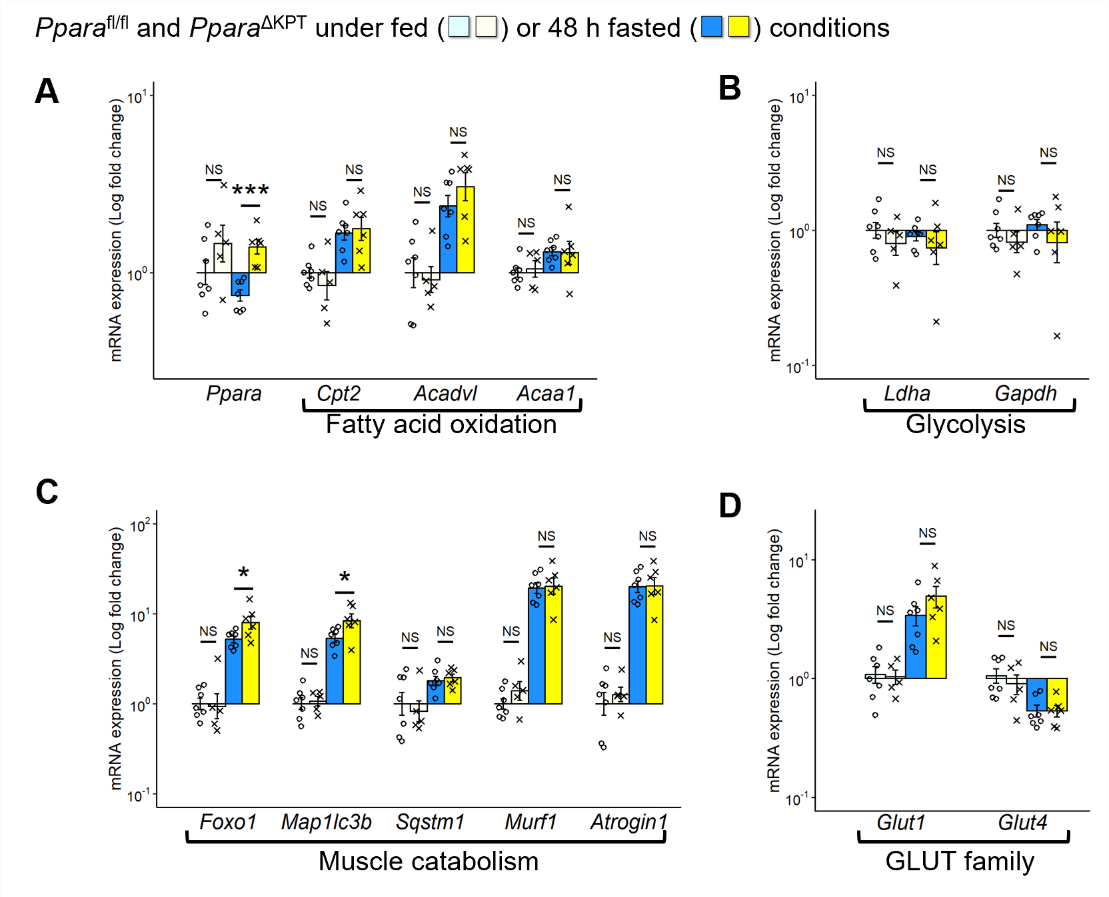
**

**Supplementary Figure 9. mRNA expression of the gastrocnemius muscles during fasting**

mRNA expression of (A) fatty acid oxidation-related genes, (B) glycolysis-related genes, (C) muscle catabolism-related genes, and (D) glucose transporters (GLUTs) in the gastrocnemius muscles. A significant difference between the groups of *Ppara*^∆KPT^ and *Ppara*^fl/fl^ controls with the same fasting time is indicated with asterisks. *: *p* < 0.05. ***: *p* < 0.001. No significant differences are indicated with NS.

*
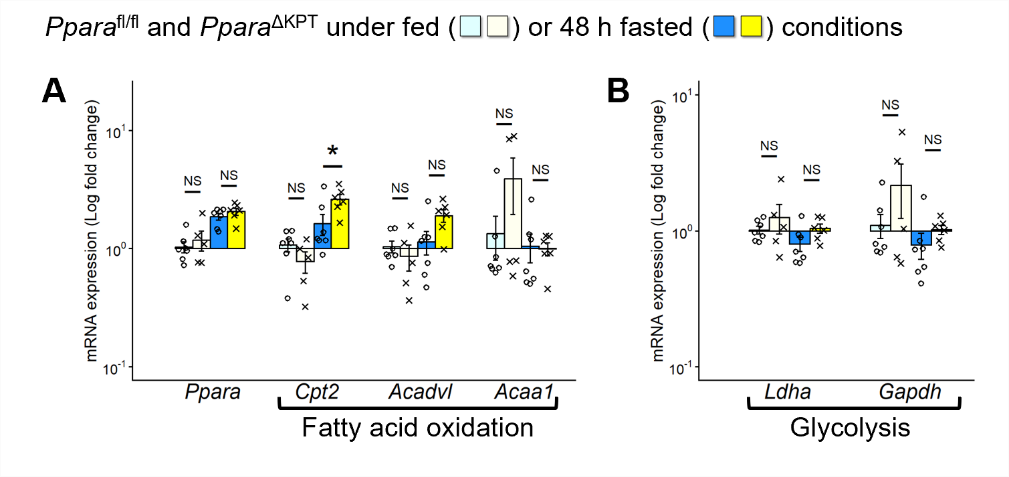
*

**Supplementary Figure 10. mRNA expression of the heart during fasting**

mRNA expression of (A) fatty acid oxidation-related genes and (B) glycolysis-related genes. A significant difference between the groups of *Ppara*^∆KPT^ and *Ppara*^fl/fl^ controls with the same fasting time is indicated with an asterisk. *: *p* < 0.05. No significant differences are indicated with NS.
